# New Research on the *Bacillus anthracis* Genetic Diversity in Siberia

**DOI:** 10.3390/pathogens12101257

**Published:** 2023-10-18

**Authors:** Vitalii Timofeev, Irina Bakhteeva, Kseniya Khlopova, Raisa Mironova, Galina Titareva, Yulia Goncharova, Viktor Solomentsev, Tatiana Kravchenko, Ivan Dyatlov, Gilles Vergnaud

**Affiliations:** 1 State Research Center for Applied Microbiology and Biotechnology (SRCAMB), 142279 Obolensk, Russia; 2 Institute for Integrative Biology of the Cell (I2BC), Université Paris-Saclay, CEA, CNRS, 91198 Gif-sur-Yvette, France

**Keywords:** anthrax, *Bacillus anthracis*, canSNP groups, Siberia and Central and Southern Russia

## Abstract

Anthrax is a particularly dangerous infection of humans and ungulates caused by the Gram-positive spore-forming bacterium *Bacillus anthracis*. The highly monomorphic and clonal species *B. anthracis* is commonly divided into three main lineages, A, B, and C, which in turn are divided into several canSNP groups. We report here a phylogenetic analysis based on the whole-genome sequence (WGS) data of fifteen strains isolated predominantly in Siberia or Central and Southern Russia. We confirm the wide distribution of the cluster of strains of the B.Br.001/002 group, endemic to the Russian Arctic, which is also present in the steppe zone of Southern Siberia. We characterize additional branches within the major A.Br.001/002 polytomy comprising the A.Br.Ames and A.Br.Sterne lineages, one of which is identified in the Arctic.

## 1. Introduction

Anthrax is a particularly dangerous disease caused by the Gram-positive bacterium *Bacillus anthracis*. This disease affects mainly ungulate herbivorous mammals but can also be transmitted to humans. The pathogenicity factors that determine the pathogenic potential of *B. anthracis* and distinguish this pathogen from closely related microorganisms are the anthrax toxin and the capsule. These factors are encoded by genes located on two plasmids, pXO1 and pXO2, called virulence plasmids. A key feature of *B. anthracis*, which largely determines its epidemiological potential and population structure, is the ability to form endospores that are extremely resistant to adverse environmental factors and able to remain viable for a long time. Infection of susceptible animals is most often initiated by spores. In the body of a susceptible animal, spores germinate into a vegetative cell, which multiplies and causes infection. After the death of the animal, vegetative cells that have entered the environment contained in fluids flowing from the corpse and/or when predators eat the corpse, will sporulate, closing the life cycle of *B. anthracis* [1,2]. This vegetative cell–spore cycle and the preservation of spores in the soil in a physiologically inactive state for decades, and possibly centuries [3], significantly reduces the rate of evolution of *B. anthracis*, because it allows a low number of replication cycles per year [4]. The high stability and long-term preservation of the viability of spores facilitated the long-distance spread of this pathogen via anthropogenic factors, including the transportation of contaminated livestock products, and also made *B. anthracis* potentially applicable for the creation of biological weapons or for bioterrorism.

*B. anthracis* displays very little phenotypic and genetic diversity. However, the advent of whole-genome sequencing allowed the development of convenient molecular typing tools. One of the most widely used methods for dividing *B. anthracis* into major phylogenetic groups is typing based on a small set of so-called “canonical” SNPs (canSNPs). Based on the canSNP profile, *B. anthracis* strains can be divided among canSNP groups into three main evolutionary lines: A, B, and C [5]. Lineage C, represented by the C.Br.001 group, is extremely rare; strains related to it are found only in North America [5,6]. Lineage A is the most genetically diverse, it combines canSNP groups A.Br.008/009, A.Br.001/002, A.Br.Ames, A.Br.003/004, A.Br.005/006, A.Br.Aust94, A.Br.Vollum, and A.Br.WNA. The strains of these groups are found all over the world, but some geographical patterns of their distribution can be distinguished [7,8]. The most widespread canSNP group is A.Br.008/009, also referred to as TEA (trans-Eurasian), whose strains are present in Eurasia and are very widely represented in Russia [9]. This group is divided into two subgroups: A.Br.008/011 and A.Br.011/009 [10,11]. A.Br.008/011 is a polytomy comprising seven radiating branches. Three of these branches have been named according to associated vaccine strains, “Pasteur”, “Tsiankovskii”, and “STI”. The Pasteur vaccine made famous by the demonstration of its efficiency at Pouilly-le-Fort (France) in 1881 comprised two strains, a low virulence “Pasteur I” belonging to A.Br.008/011 and a higher virulence “Pasteur II” strain belonging to A.Br.011/009. Tsenkovsky developed a similar vaccine in 1883–1887 following the approach invented by the Pasteur team [12]. The second strain constituting these first-generation vaccines still contained both virulence plasmids and Pasteur II for instance has kept some zoonotic potential, as shown in Vietnam [13]. In addition, the residual virulence of the Tsenkovsky vaccine is sufficient to use this strain as a challenging strain to evaluate the effectiveness of other anthrax vaccines for laboratory animals [14]. Group A.Br.001/002, which is similarly structured to a polytomy, is widespread in China (central and eastern regions). From here came the A.Br.Ames group, brought from Asia, possibly China, to the USA [15,16]. The A.Br.WNA and A.Br.003/004 groups are widely distributed in North and South America, respectively. The A.Br.005/006 group is found in Australia, Africa, and Europe; the A.Br.Aust94 group is common in Southeast Asia, India, Australia, Western China, Turkey, and Caucasus, the A.Br.Vollum group is found in South Africa and some regions of Asia (Pakistan and Afghanistan) [7,17].

Over the past 10 years, the remarkable decrease in whole-genome sequencing (WGS) costs has allowed WGS to progressively be applied to ancient strain collections (retrospective investigations) as well as recent outbreaks. The wealth of available WGS data covering all known lineages confirms the very low level of genetic diversity and the strict clonality established by pioneering investigations [6]. The current view is that the low diversity reflects the recent emergence of the pathogen [4,5,16,18,19]. However, dating is made challenging due to the variable speed of evolution caused by the dormant state. There is currently a single robust dating point on the phylogeny, the post-Columbian dating of the export of the WNA lineage predominant in North America [4,18,20]. This single dating point combined with indirect evidence inferred from its geographic distribution provide indications regarding the emergence of lineage A.Br.008/009 [16]. Unfortunately, the absence of a global molecular clock makes it impossible to extrapolate dating points for the other lineages from this estimate. Such estimates will require the identification and interpretation of other remarkable features along the phylogeny.

The clonality of *B. anthracis* phylogeny implies that the species emerged at one time in one geographic location. The current best evidence in favor of an African origin for *B. anthracis* is the finding in Africa of *Bacillus cereus* strains carrying both virulence plasmids [21,22]. One proposed working hypothesis is that the Ancient A clade alias A.Br.005/006 [10], which has an almost exclusively African association, is the modern representative of the original ecotype, and that the C, B and A branches represent successive out-of-Africa exports [16,19]. The distribution area of lineage B and its genetic diversity are significantly more restricted than those of lineage A. Lineage B includes three canSNP groups: B.Br.CNEVA, present in Western and Central Europe, B.Br.Kruger, found in South Africa, and B.Br.001/002, strains of which until recently were thought to be found only in South Africa and Korea [23,24]. During the past years, B lineage was shown to be present in a much wider area. The anthrax outbreak on the Yamal Peninsula in 2016 was due to a B.Br.001/002 group strain [3]. Another B.Br.001/002 lineage was identified in Finland [24]. Subsequently, the Stavropol *B. anthracis* investigation group described strains from Central and Southern Russia and Siberia, defining new sublineages within B.Br.001/002 [9,25,26]. A genetically and geographically closely related strain was isolated in the north of Kazakhstan [16]. Thus, the B.Br.001/002 group turned out to be endemic for Central Eurasia, and its distribution area extends for at least 2000 km from north to south and from west to east.

These examples illustrate that the description of the biodiversity of the anthrax microbe in Russia and Siberia might help to better understand its phylogeography. In this respect, Siberia is understudied, mainly due to the extremely low population density and poorly developed infrastructures. The low population density in Siberia is due to the unsuitability of most of its territory for economic activity. This is especially pronounced in the northern part, most of which is located in the permafrost zone. Global warming might favor changes. In this case, two potentially dangerous processes can occur: (1) thawing of pathogens including *B. anthracis* frozen in the permafrost and (2) an increase in the number of people and animals susceptible to anthrax in the region. Together, these processes can lead to epidemics and epizootics of anthrax. Knowledge of which strains are currently endemic in Siberia will facilitate epidemiological investigation of such potential outbreaks.

In this report, we present the results of a phylogenetic analysis of fifteen strains, four of which originate from Siberia. We use the associated WGS data to anchor part of the Stavropol collection using the published list of SNPs, since the corresponding WGS data were not deposited in public repositories [9,25,26].

## 2. Materials and Methods

### 2.1. Bacterial Culture, Strain Collection, DNA Extraction, and PCR Analyses

BHI agar (SRCAMB, Obolensk, Russia) was used for bacterial cultivation. All manipulations with live strains were performed in a biosafety laboratory level 3. The list of strains used in this study is provided in Table S1.

Total DNA was isolated using the “Genomic DNA Purification” #Kit K0512 (Thermo Fisher Scientific, Waltham, MA, USA). The sterility of the DNA samples was verified by cultivating 5% of the extracted DNA solution on agar plates. The DNA concentration was quantified using the NanoDrop One spectrophotometer (Thermo Fisher Scientific).

PCR amplifications were run on the CFX96 Real-Time PCR Detection System (Bio-Rad, Moscow, Russia). The “OM-screen-anthrax-RT” test system (Syntol, Moscow, Russia) was used to detect the pXO2 plasmid. For multiple loci VNTR (variable number of tandem repeats) analysis (MLVA) and canSNP genotyping, 2.5× PCRmix M-427 with SYBR-GreenI (Syntol, Moscow, Russia) were used. The PCR primers were synthesized by Syntol, Russia.

MLVA was performed using published primers [5,27,28,29]. Seventeen loci compatible with agarose-gel typing given their repeat unit and overall allele size were used: vrrA, vrrB1, vrrB2, vrrC2, bams01, bams03, bams05, bams21, bams22, bams23, bams24, bams25, bams28, bams34, bams44, bams51, and VNTR23. We also used the bi-allelic locus VNTRacpA, which differentiates lineages A (3 repeat units) and B (4 repeat units) [30,31]. Monoplex PCR products and a 20 bp ladder (Bio-Rad, Hercules, CA, USA) were electrophoresed at 100 V for four hours on a 32 cm length 3% agarose gel prepared in 0.5× TBE. The DNA fragments were visualized with ethidium bromide staining and ultraviolet light (312 nm) using the Doc-Print gel documenting system and PhotoCaptMw software version 99.04 (Vilber Lourmat, Marne-la-Vallée, France). PCR products larger than 600 bp were reanalyzed on 2% agarose gel for better resolution.

### 2.2. Draft Whole-Genome Sequencing (WGS)

Sequencing was performed on a DNBSEQ-G400 platform (MGISEQ-2000) (MGI Tech, Wuhan, China). DNA libraries were prepared using the MGIEasy Universal DNA Library Prep Set. Fragmentation was carried out in a Bioruptor ultrasonic DNA fragmentation system (Diagenode, Denville, NJ, USA). The whole-genome sequencer was run using the DNBSEQ-G400RS High-throughput Sequencing Set (FCL PE150) (2 × 150 bp) according to the manufacturer’s recommendations. Raw sequencing data were deposited in Bioproject PRJNA1010109, accessible at https://www.ncbi.nlm.nih.gov/bioproject/1010109 (accessed on 28 September 2023). Individual sequence reads archives (SRA) accessions are also indicated in Table S1.

### 2.3. Data Analysis

Single-nucleotide polymorphisms (SNPs) were called by mapping raw sequencing reads on an Ames ancestor reference genome assembly accession GCF_000008445.1 (including chromosome and pXO1-pXO2 plasmid accessions NC_007530.2, NC_007322.2, and NC_007323.3,). BioNumerics version 8.1 (Applied-Maths, Laethem-Saint-Martin, Belgium) was used for SNP calling as previously described, with a minimum 5× coverage [3]. For comparison, publicly available assemblies and raw reads were downloaded via EBI-ENA (last update 2 December 2022). Assemblies were split into 50 bp long artificial reads that were then used for SNP calling. Lineage assignments followed the global nomenclature based on selected SNPs [10]. BioNumerics was used for maximum parsimony or UPGMA analysis and dendrogram drawing.

## 3. Results

### 3.1. Selection of Strains

Fifteen strains were available to this project. Four originated from Siberia, one from Mongolia and six from Caucasia, Central Russia, or the Caspian steppes. Two strains originated from Lithuania and the geographic origin of the last two is unknown (Table S1). All these strains are typical, except for Bac4 from the Yamal peninsula. When sown on a nutrient medium, Bac4 formed only R-form colonies, which is typical for non-capsular strains. Microscopic examination confirmed the absence of a capsule, and PCR analysis showed that this strain lacks the pXO2 plasmid. Unfortunately, data on its plasmid profile at the time of isolation were not preserved, so we cannot state whether the plasmid was lost during storage or whether the strain was isolated without this plasmid.

### 3.2. MLVA Typing

**Figure 1:**
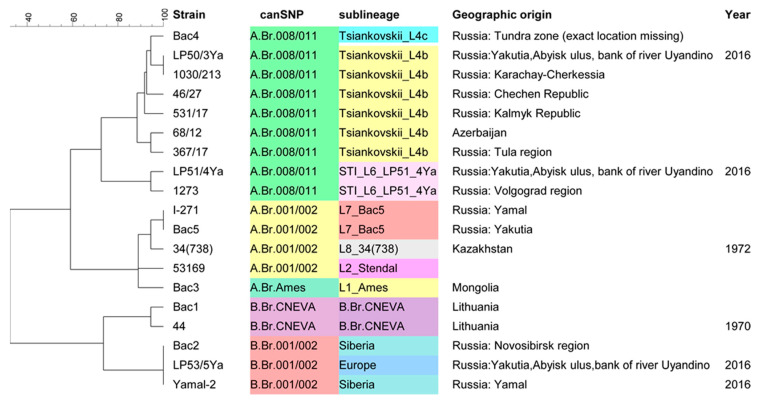
UPGMA dendrogram deduced from MLVA data and illustrating the clustering of the 15 strains described in this article and four strains isolated earlier in the Arctic (described in [3]). Clustering analysis was performed using BioNumerics. The canSNP and sublineage assignments subsequently deduced from wgSNP analysis are shown to illustrate the congruence with MLVA clustering. The MLVA profiles are shown in Table S1.

MLVA was used as a first-line assay (Figure 1 and Table S1). This typing method allows fast and accurate typing of *B. anthracis* strains with high discriminatory power. Strains Bac1, Bac2, and 44 belong to the B lineage. Strain Bac1 (isolated in Lithuania) has almost the same MLVA profile as strain 44 (canSNP group B.Br.CNEVA), differing from it in a single locus. At the time of publication of previous articles mentioning strain 44 [32], information about the isolation point was considered lost. However, later, we found an entry in old work logs indicating that this strain had been isolated in Lithuania. Despite the fact that these data are not completely official and reliable, the high similarity of the MLVA profile of strains 44 and Bac1 provides evidence to confirm the place of isolation of strain 44.

Strain Bac2, isolated in the vicinity of Novosibirsk, has the same MLVA profile as two B.Br.001/002 strains, Yamal-2, responsible for an anthrax outbreak in Yamal in 2016, and KZ178, isolated in Northern Kazakhstan in 2016 in the vicinity of Pavlodar, approximately 400 km southwest of Novosibirsk [3,16]. Geographically, Pavlodar and Novosibirsk are located in Southern Siberia. Thus, our previously published data, the data published in [16], and the results published in this article together show that closely related strains of line B are distributed in the center of Eurasia, which is in agreement with previous reports [9]. Their distribution area is more than 2000 km in diameter and covers both the Arctic and the south of Siberia on the border of the steppe zone.

Of the twelve other strains belonging to the A clade, Bac5, isolated in Yakutia, has the same MLVA profile as strain I-271 (canSNP group A.Br.001/002), isolated in the Yamalo-Nenets region. The similarity of the A-clade strains isolated in Yamal and Yakutia is very interesting given the distance of about 1000 km between the administrative boundaries of these regions. It is also reminiscent of the similarity of B-clade strains previously recovered in Yamal and Yakutia [3,9]. Thus, in addition to the Siberian cluster of strains of the B.Br.001/002 group, we may have evidence of the existence of a cluster of strains of the A.Br.001/002 group circulating in Siberia.

### 3.3. Whole-Genome Sequence Analysis

To obtain more informative data, including those showing the difference between strains with the same MLVA profile, we performed whole-genome sequencing for the fifteen strains.

### 3.4. canSNP Group Determination

In agreement with the MLVA assignments, Bac1 and strain 44 belong to the B.Br.CNEVA group and Bac2 belongs to the B.Br.001/002 group. Four strains belong to A.Br.001/002 and one to A.Br.Ames. The last seven belong to the A.Br.008/011 group (Figure 1 and Table S1).

### 3.5. Linking the Present Collection with the Stavropol Collection SNP Data

The Stavropol group published a list of SNPs identified among a collection of 66 strains collected in the Former Soviet Union (FSU) and a number of public WGS datasets [9]. This collection represents the most extensive description of Russian strains to date; however, the lack of the WGS data itself makes comparisons more difficult. In order to compare the genotypes of these strains with the present collection of fifteen strains and four strains from a previous report [3], we called SNPs using the same reference genome, Ames Ancestor chromosome accession NC_007530 (corresponding to the GCF_000008445 assembly minus the two plasmids). The table of 1645 SNPs was then merged with the Eremenko et al. SNP dataset [9] and analyzed using the UPGMA clustering method (Figure S1). There is a good overlap between the two collections within the A.Br.001/002, A.Br.008/011, and B lineages. Duplicate strains I-271 and I-271_OBL are coincident as expected.

### 3.6. Comparison with Public WGS Datasets by Whole-Genome SNP (wgSNP) Analysis

**Figure 2:**
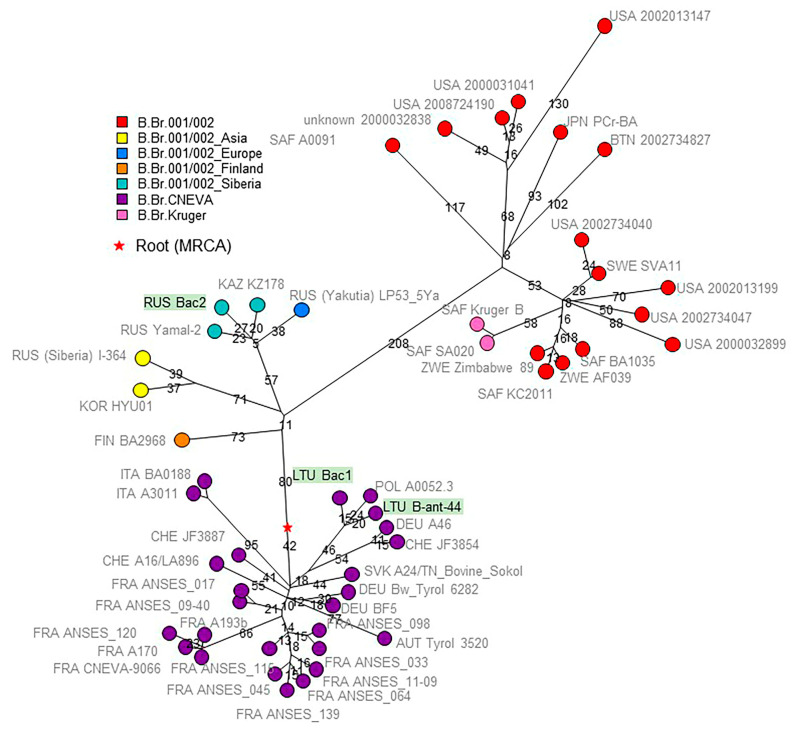
Topology of the B clade. Fifty-one strains were included, 2724 SNPs were called, and the maximum parsimony tree size is 2726 (homoplasia 0.07%). The red star indicates the position of the root or MRCA of the lineage. Nodes are colored according to lineage as previously defined [9]. Nodes are labelled with three-letter country codes and strain ids. The three strains from the current investigation are underlined in green. Branch lengths above four SNPs are indicated.

wgSNP analysis allowed us to precisely locate strains Bac1, Bac2, and 44 within the B clade (Figure 2). It is interesting that the area of distribution of the Siberian group of strains is represented by two subareas—the steppe zone of Eurasia in the south and the tundra in the Arctic. These subareas are separated by a zone of temperate forests and boreal forests, offering limited opportunities for the natural circulation of *B. anthracis*.

Strains Bac1 and 44 from Lithuania clustered together with the strain from Poland within the B.Br.CNEVA group. Figure 2 illustrates the clear geographic separation between the B.Br.CNEVA lineage found so far only in Western Europe, with Poland, Lithuania, and Slovakia as current most eastern locations and B.Br.001/002 present in Western Russia and Siberia. Bac2 from the Novosibirsk region belongs to the lineage called “Siberia” [9], which also contains strain I-271_IRK from Yakutia (Figures S1 and S2). The “Finland” branch in Figure 2 was populated by strains from Southern Siberia (Omsk region) (Figure S1) [9]. The phylogenetic tree of the B branch contains a remarkably long branch, reminiscent of the long branch leading to the North American WNA lineage [18]. This branch leads to sublineages represented by South African strains, including the Kruger branch, but also by strains from Eastern Asia, including Bhutan, Thailand, and Japan. This strongly suggests that the long branch expansion occurred in the Eurasia continent before the B lineage was exported to South Africa. Two independent introductions to South Africa are required to explain the observed topology. These events must have occurred in the past few centuries, after the establishment of the appropriate maritime trade routes. More data from Southeast Asia will be needed to better pinpoint these events on the B-branch phylogeny.

**Figure 3:**
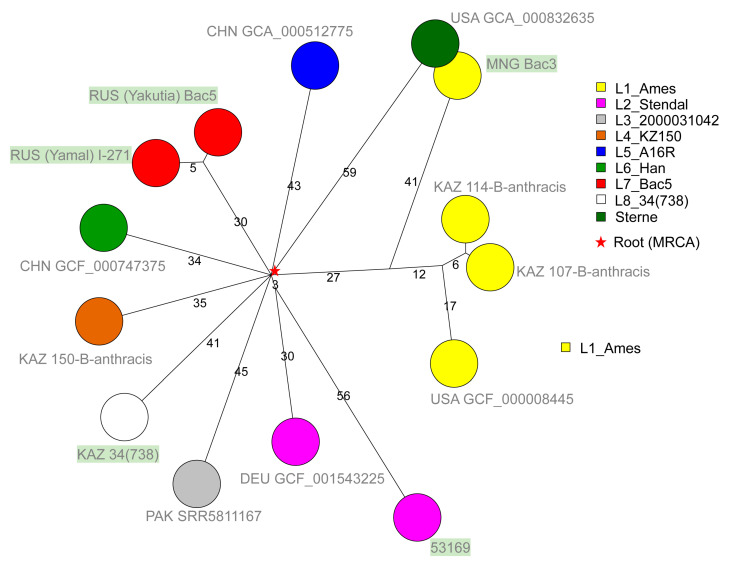
Topology of the A.Br.001/002 group. The group is a polytomy with nine branches, including the Sterne and Ames clades. Nodes are labelled with the three-letter country code and strain ids. In addition to the five strains from the present investigation labelled in green, one to three (L1_Ames lineage) representative strains from each lineage are included. The red star indicates the position of the root or MRCA of the lineage. It is located one SNP from the center of the polytomy along the Sterne branch. A total of 489 SNPs were called among the fourteen strains. The resulting maximum parsimony tree has a size of 489 (no homoplasia). Branch lengths above two are indicated.

**Figure 4:**
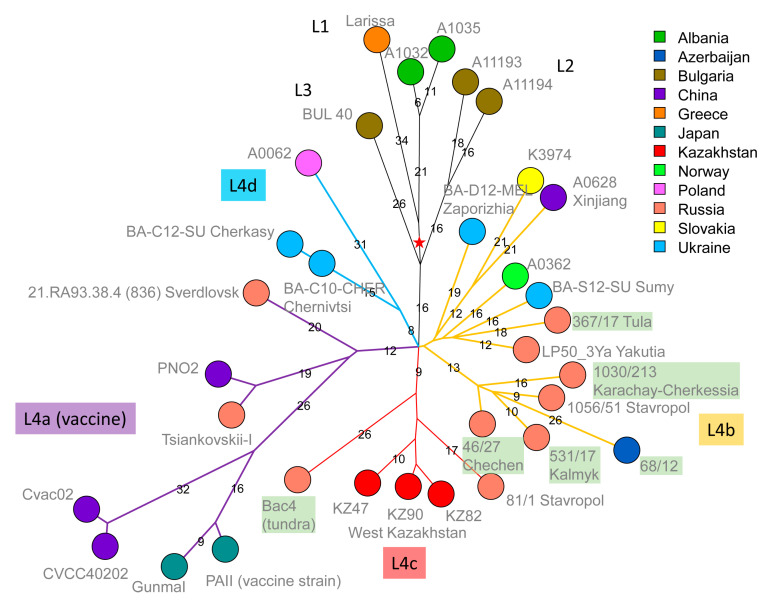
Topology of the Tsiankovskii lineage. A total of 688 SNPs were called among the 33 strains assigned to the A.Br.008/011 Tsiankovskii lineage. The resulting maximum parsimony tree has a size of 689 (homoplasia level 0.14%). Nodes are colored according to country of origin and labelled with the strain id followed by the region of isolation (when available). The labels of the six strains from the present investigation are colored in green. The red star indicates the root (position of the most recent common ancestor) of the Tsiankovskii lineage. Branches and lineage labels within the L4 sublineage are colored for clarity. Branch lengths above five SNPs are indicated.

The result of phylogenetic analysis of A-lineage strains is shown in Figure 3 and Figure 4.

The five strains assigned to the A.Br.001/002 group populate four of the nine branches constituting the A.Br.001/002 polytomy. Bac3 from Mongolia belongs to branch L1, which also contains the Ames sublineage [21]. Strain 53169 (of unknown geographic origin within the FSU) belongs to L2_Stendal. Bac5 from Yakutia is closely related to strain I-271, which was isolated in the Yamalo-Nenets region, and the two strains define lineage L7. Strain 34(738) from Kazakhstan defines branch L8. This lineage is also present in Southern Siberia (Omsk) and Altai (Figure S1) [9]. The A.Br.001/002 polytomy is currently the most remarkable polytomy within *B. anthracis* regarding the number of branches.

Six strains belong to the Tsiankovskii lineage of the A.Br.008/011 polytomy [21]. Figure 4 shows the Tsiankovskii phylogeny based on the wgSNP data from 33 strains. Four sublineages, labelled L1 to L4, were previously defined [11]. Lineages L1 to L3, derived from the most ancestral nodes, are represented by strains from Bulgaria, Greece, and Albania. Lineage L4 is a polytomy with four branches, L4a to L4d. L4a contains the Tsiankovskii-I vaccine strain itself, together with a collection of vaccine strains presumably derived from the historical Tsiankovskii vaccine strains. For instance, vaccine strain PNO2 (alias Qiankefusiji II) was introduced from Russia to China in 1956 [33]. The genome assemblies of all these vaccine strains contain the virulence plasmids, except for the Cvac02 assembly GCF_000747335 that lacks both. Five strains from the present investigation are assigned to L4b. Four, including the strain from Azerbaijan, constitute a Caucasia lineage. The fifth belongs to a geographically more diverse group, including two strains from Eastern Ukraine. Bac4 (of uncertain geographic origin) belongs to L4c, which is also populated with strains from Western Kazakhstan [16]. Bac4 is coincident with strain 881-1 from the Astrakhan region (Eremenko et al. collection, Figure S1), strengthening the proposition that L4c was introduced into Kazakhstan from Russia via the north of the Caspian Sea [9,16].

The last strain from the present collection, strain 1273, was assigned to STI lineage 6 [16]. This strain, from the Volvograd region, is closest to strains from Dagestan and West Kazakhstan (Figures S1 and S2).

## 4. Discussion

In the present report, we described the phylogenetic position of fifteen newly sequenced strains from the FSU by comparison with publicly available sequence data. Using wgSNP analysis, the strains could be precisely assigned to four lineages, A.Br.001/002, B.Br.CNEVA, B.Br.001/002, and A.Br008/011 (five, two, one, and seven strains, respectively). In particular, the biological diversity of *B. anthracis* in Siberia turned out to be significantly greater than estimated ten years ago. The extremely low population density (0.36 people per square kilometer in Yakutia and 0.7 people per square kilometer in the Yamalo-Nenets District), the lack of the possibility of developing land transport infrastructure, as well as the peculiarities of the nomadic pastoralism practiced in this region has caused an insufficiency of epidemic surveillance over this territory. The situation changed somewhat after the anthrax epidemic in Yamal in 2016, which clearly showed the lack of up-to-date information on *B. anthracis* strains endemic to Siberia [3,9,25,26].

The A.Br.001/002 group is structured as a remarkable polytomy comprising nine branches. One of the two most recently described branches is defined by three strains from Southern Siberia, Altai, and Kazakhstan. The second new branch is defined by two genetically very close strains, one isolated in the Yamal peninsula and the other in Yakutia, locations separated by more than 1000 km but located in the same ecosystem (Arctic tundra) and not separated by natural barriers that prevent the migration of animals susceptible to anthrax (reindeer). This opens the possibility that these strains represent a genotype circulating in the Arctic. Alternatively, these two strains might have been independently introduced to Yakutia and Yamal from an unknown region via anthropic factors, such as the transport of contaminated animal products.

A similar geographic distribution is observed within B.Br.001/002, with high genetic proximity between strains from Southern Siberia, the Yamal peninsula, and Yakutia, which might also be the result of anthropic activities [3,9]. The Ob, Yenisei, and Lena rivers and their tributaries can be “anthropogenic” vectors of strain transfer. These rivers flow from the south (from the forest-steppe zone) to the north through the taiga and tundra. In the absence of a road network, rivers have long been used as transport routes both in the warm season for sailing and in winter as a sledge track. Starting from the 16th century with the Russian colonization of Siberia, the role of such river routes in ensuring the transport connectivity of the continent has grown significantly, increasing the likelihood of the spread of strains of pathogenic microorganisms, including *B. anthracis*. Starting from Southern Siberia, the Ob route would connect to the Yamal peninsula, whereas the Lena route would connect to Yakutia. Distinct *B. anthracis* lineages originating from Western Eurasia (the B clade) or Central and Eastern Eurasia (the A.Br.001/002 clade) could have been transferred to very remote areas via similar routes.

Seven among the fifteen strains investigated here belong to A.Br.008/011, alias TEA [21]. Similarly to A.Br.001/002, this lineage is structured in a polytomy, which comprises seven branches. Six of the strains are assigned to the Tsiantovskii branch, which is increasingly populated by strains from Western Russia, particularly North Caucasia. The phylogeny of the Tsiankovskii branch suggests that it spread from Turkey via Bulgaria along the northern part of the Black Sea and Caspian Sea.

## 5. Conclusions

We report that the Arctic B.Br.001/002 group, endemic to the Russian Arctic, is also present in the steppe zone of Southern Siberia. Thus, the distribution area of this group turned out to be much larger than previously thought. We also describe a new branch within the A.Br.001/002 polytomy, which is similarly distributed in the Arctic. Whole-genome sequence data from many more strains will be needed to understand the spread of the anthrax microbe in Siberia and in the world in general, but some robust patterns are beginning to emerge.

## Data Availability

All data used for this study are available in the text of the article and in the Supplementary Materials.

## Supplementary Materials

The following supporting information can be downloaded at: https://www.mdpi.com/article/10.3390/pathogens12101257/s1. Table S1. List of strains used in this study. Figure S1. Whole-genome SNP-based UPGMA-dendrogram combining a set of strains from this study with a set of strains described in [9]. Figure S2. Topology of the STI lineage strains used in this study.
