# Supplementary figures and images for: New Research on the Bacillus anthracis Genetic Diversity in Siberia
Source: Pathogens. 2023 Oct 18;12(10):1257. doi: 10.3390/pathogens12101257 (PMC10610006; doi:10.3390/pathogens12101257)

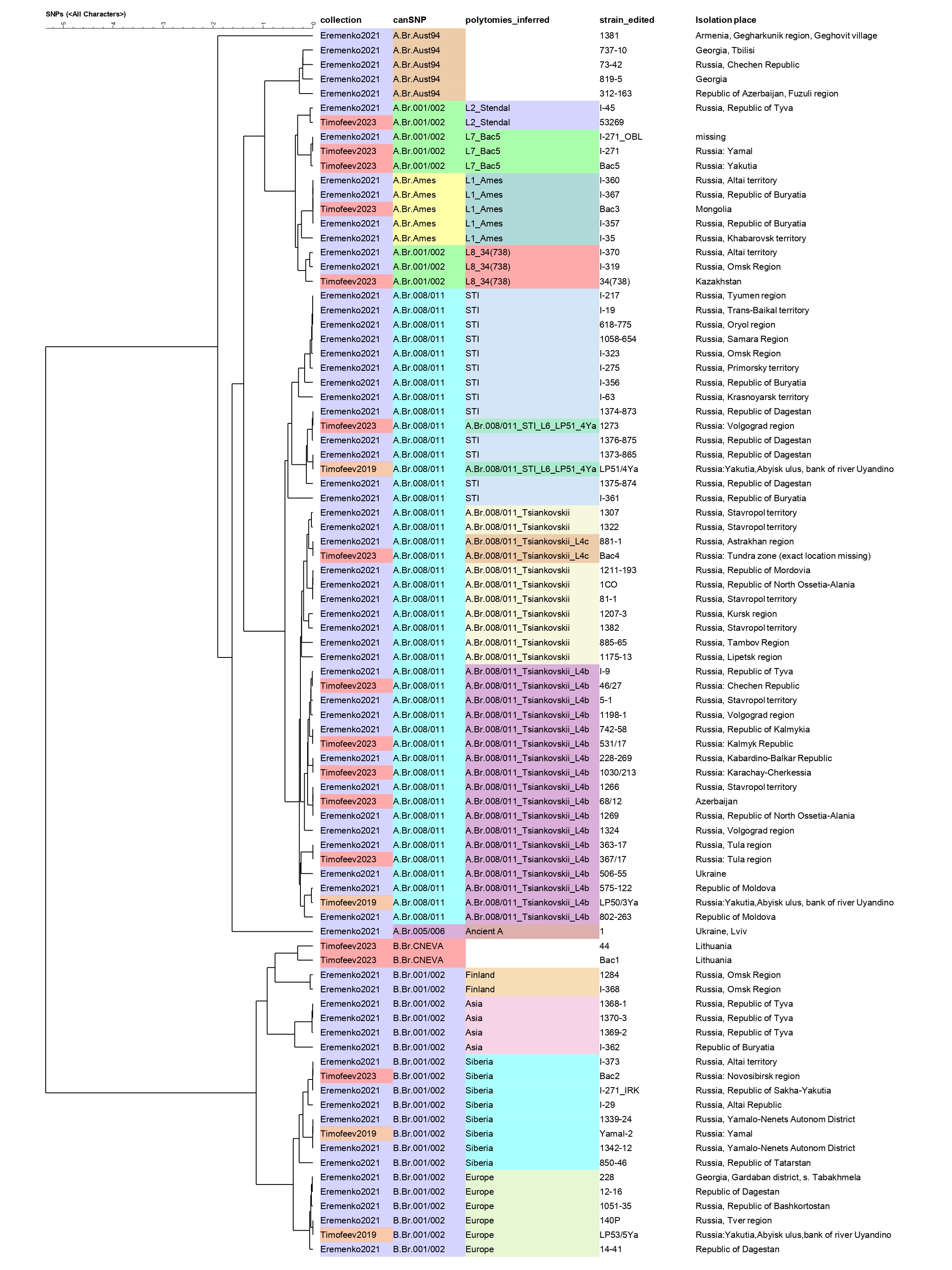

Supplement: Supplementary file 1 [file pathogens-12-01257-s001.zip › Figure S1.jpg]

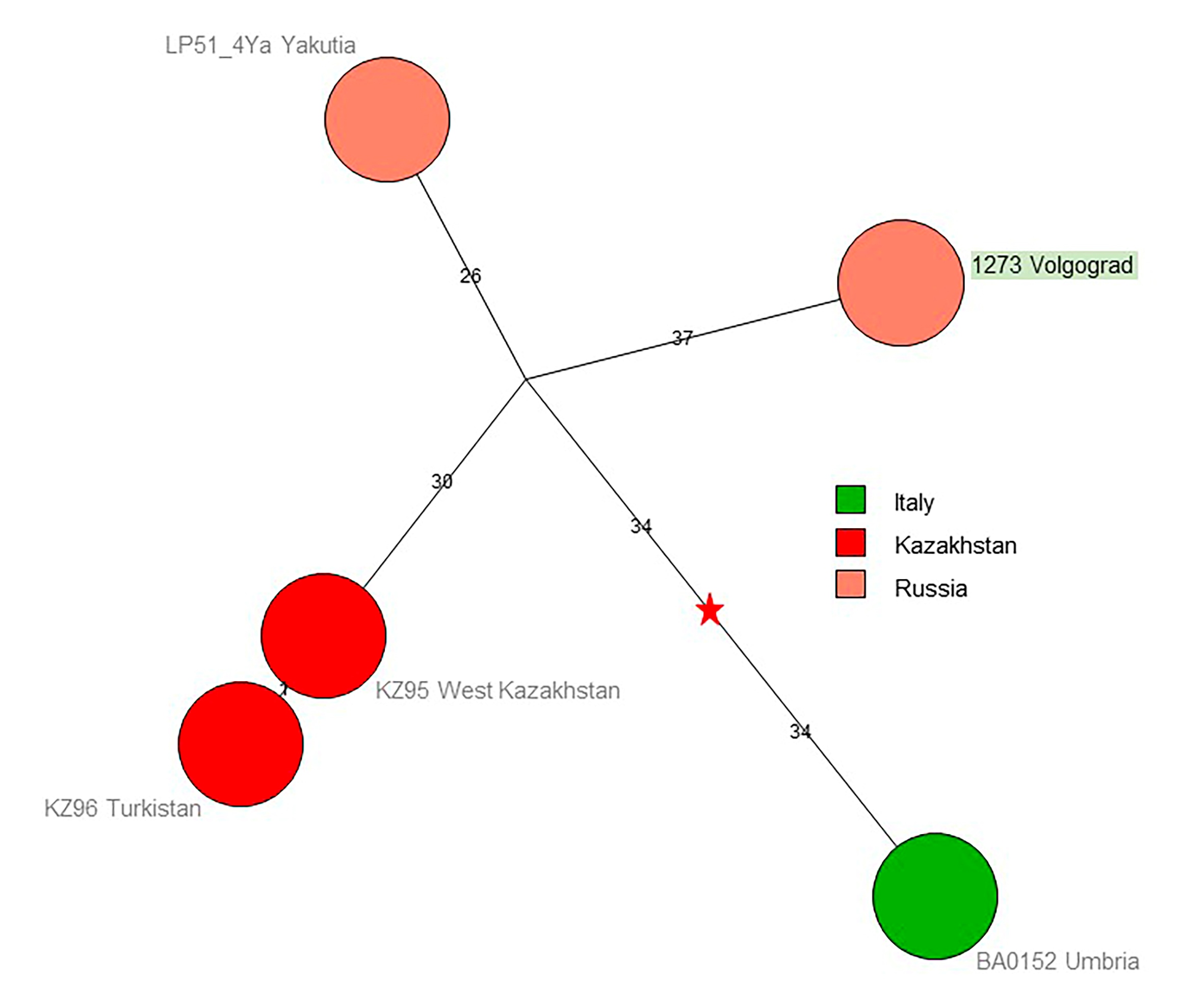

Supplement: Supplementary file 1 [file pathogens-12-01257-s001.zip › Figure S2.jpg]
